# Exploring the motives for drinking less alcohol and attitudes towards abstinence in individuals with low-to-moderate alcohol use – a mixed-methods study

**DOI:** 10.1186/s12889-025-26015-7

**Published:** 2026-01-07

**Authors:** Maria Zeiser, Sophie Baumann, Jennis Freyer-Adam, Antje Ullrich, Ulrich John, Andreas Staudt

**Affiliations:** 1 Institute and Policlinic of Occupational and Social Medicine, Faculty of Medicine, TU Dresden, Fetscherstraße 74, Dresden, D-01307 Germany; 2 Department of Methods in Community Medicine, Institute for Community Medicine, University Medicine Greifswald, Greifswald, Germany; 3 Department of Prevention Research and Social Medicine, Institute for Community Medicine, University Medicine Greifswald, Greifswald, Germany; 4 Department SHIP/Clinical-Epidemiological Research, Institute for Community Medicine, University Medicine Greifswald, Greifswald, Germany

**Keywords:** Alcohol use, Drinking motives, Low-to-moderate drinking, Abstinence, Non-drinking, Mixed-methods, Social norms, Cultural norms

## Abstract

**Background:**

Extensive evidence links alcohol to increased morbidity and mortality, leading to recommendations to reduce drinking, regardless of the amount consumed. We aimed to explore the motives for (not) drinking alcohol in low-to-moderate drinkers because of their relevance for prevention strategies.

**Methods:**

Using a mixed-methods design, we first analyzed longitudinal self-report data from 540 low-to-moderate drinkers from general population in Germany who participated in a randomized controlled trial. Over a period of three years, trajectories of alcohol use were examined using latent class analysis, resulting in the identification of three distinct classes with similar patterns of change. To gain deeper insights in (non-)drinking motives, we conducted qualitative interviews with 22 individuals sampled from these classes (55% female, mean age = 36 years, *SD* = 13.2). Interview transcripts were analyzed using content-structuring qualitative content analysis.

**Results:**

Social motives dominated across all trajectory classes. Enhancement and conformity motives were also present, coping motives appeared rarely. Several participants offered fragmented, hesitant, or contradictory reasons for drinking, which illustrated their habitual and automated drinking patterns or unreflective routines. Impression management, as well as health-related beliefs, appeared as additional motivational dimensions. Situational abstinence was common, yet sustained abstinence faced barriers such as the cultural normalization of drinking, the low perceived health risks of light drinking, and the avoidance of the social costs of abstinence. Participants with the lowest consumption in our sample highlighted autonomy-related motives for abstaining, while higher-consuming groups described conformity and social adjustment more frequently.

**Conclusions:**

Even within a narrow range of low-to-moderate drinking patterns, motives, resources, and barriers differ meaningfully. These findings highlight the need for prevention strategies that acknowledge cultural norms, social contexts, and motivational heterogeneity beyond heavy drinking populations.

**Trial registration:**

The study was preregistered on the Open Science Framework, reviewed and approved by the ethics committee of the University Medicine Greifswald (BB 034/22), and conducted in accordance with the Declaration of Helsinki. All participants provided written informed consent prior to participation, including consent for the anonymized publication of their data.

**Supplementary Information:**

The online version contains supplementary material available at 10.1186/s12889-025-26015-7.

## Background

Extensive evidence shows that any level of alcohol use is associated with an increased disease risk and a shorter time to death compared to lifetime abstinence [1–7]. The protective effects of low-to-moderate alcohol use are largely explained by misclassification and confounding factors, such as a history of dependence or comorbid smoking among abstainers [2–5]. Systematic analyses also reveal industry-funded biases in research reporting cardiovascular benefits, raising concerns about conflicts of interest [8].

In light of these findings, current public health guidelines emphasize that no level of alcohol use is entirely risk-free [1, 4]. Brief alcohol interventions, particularly personalized motivational feedback, have demonstrated effectiveness among individuals with risky patterns of use [9–12], such as individuals identified as hazardous drinkers using the Alcohol Use Disorders Identification Test (AUDIT; scores ≥ 8) [13]. However, their applicability for individuals with lower levels of alcohol use has received comparatively little attention. This is partly due to limited knowledge regarding their motives, attitudes, and intentions toward non-drinking.

Research on drinking behavior has traditionally focused on motives for alcohol use, utilizing frameworks such as Cox and Klinger’s motivational model [14], further operationalized by Cooper [15]. According to Cox & Klinger (1988), drinking motives can be categorized along two dimensions: (1) the source of reinforcement (internal vs. external), and (2) the type of reinforcement (positive vs. negative) [14]. Combining these dimensions yields four well-defined motive types: (1) enhancement (internal, positive reinforcement, e.g., drinking to feel good), (2) coping (internal, negative reinforcement, e.g., drinking to manage stress), (3) social (external, positive reinforcement, e.g., drinking to enjoy social gatherings), and (4) conformity (external, negative reinforcement, e.g., drinking to avoid social rejection) [14, 16]. Over the past years, the literature has further refined this structure, for instance, by differentiating between coping with anxiety vs. coping with depression [17, 18]. Additionally, research demonstrates that drinking motives are the most proximate factor mediating the relationship between more distal factors, such as alcohol expectancies and personality traits, and alcohol use [19–22]. Motives have also been shown to interact with alcohol-related contexts to influence drinking patterns [20–22].

Drinking motives are not merely descriptive but predictive of distinct drinking patterns. Coping and enhancement are most consistently linked to risky alcohol use [16, 20, 21, 23–27]. Findings on social motives are more mixed. Many studies have reported that social motives tend to be associated with frequent drinking in social settings, though not necessarily with risky alcohol use [16, 24]. Other research has found strong associations with risky drinking in student population [26, 28]. Conformity motives show the weakest and most inconsistent correlations with drinking patterns [23–26, 28] These motives are referenced in relation to moderate alcohol use in certain social situations [15, 29] and have even been shown to have a protective effect against alcohol use [25]. The evidence may remain complex because different studies use different measurement instruments, definitions of drinking patterns, sample characteristics, and associations may vary by age group and cultural context [22, 28].

In parallel, research increasingly focuses on non-drinkers, their motives, and how they are perceived by others, as well as how they manage social occasions, in which alcohol is typically consumed [30–35]. Non-drinkers reported diverse reasons for abstaining from alcohol, including dislike of the taste or effects of alcohol, as well as lifestyle, religious reasons, or a desire to be themselves [34, 35]. Motivations vary by abstainer type: No interest in drinking and disliking the effects of alcohol were reported by lifelong abstainers; no interest in drinking and health problems or concerns were main non-drinking reasons for current abstainers; health problems or concerns were most frequently mentioned by former problem-drinkers [35].

However, motives alone do not fully explain drinking behavior or the complex process of change. Whether individuals can reduce or stop alcohol use depends on their perceived resources and barriers to change. These constructs are central to many health behavior change theories, including the Transtheoretical Model, which emphasizes the significance of perceived barriers and self-efficacy in facilitating behavioral transitions [36–38].

Little is known about the specific motivational processes underlying alcohol reduction among low-to-moderate drinkers. Unlike abstainers or risky drinkers, this population occupies a unique position: Their current alcohol use remains within traditional “low-risk” thresholds, yet still carries health risks according to the current evidence [1–7]. This creates a gray area in which health concerns seem distant and abstract or even unknown despite being real, potentially reducing openness to behavior change interventions. Since this group represents a substantial portion of adult drinkers [39, 40], understanding their motivational processes could inform more effective targeted prevention strategies.

This study uses a mixed-methods design to explore the motives (not) to drink, as well as resources and barriers to drinking reduction among German adults with low-to-moderate alcohol use. The application of a mixed-methods design allows quantitative measures to identify drinking patterns, while also capturing nuanced reasoning and contextual factors through qualitative methods.

## Methods

### Sequential explanatory design

The study and the interview guide (see Supplement) was preregistered on the Open Science Framework [41], reviewed and approved by the ethics committee of the University Medicine Greifswald (BB 034/22), and conducted in accordance with the Declaration of Helsinki. All participants provided written informed consent prior to participation, including consent for the anonymized publication of their data.

This study used an explanatory sequential mixed-method design which incorporated quantitative and qualitative approaches in two consecutive phases within one study (see Table 1). The quantitative method was conducted in the first stage and used data from the control group of a randomized controlled trial entitled “PRoactive expert system INTervention to prevent and to quit at-risk alcohol use” (PRINT [42]). Subsequently, a qualitative study was conducted using qualitative content analysis of transcripts of semi-structured interviews.

**Table 1.**
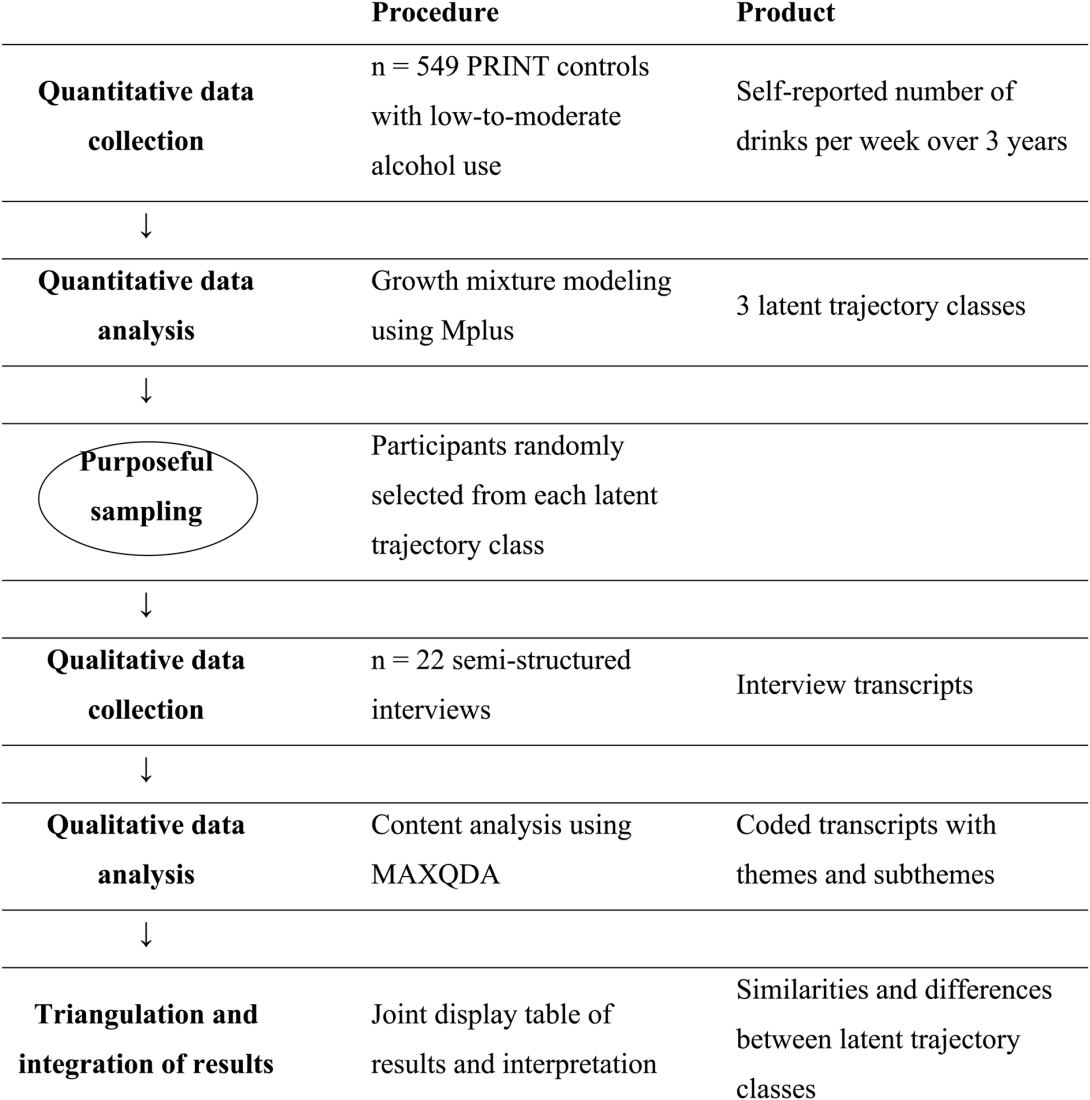
An overview of the explanatory sequential mixed-method design

### Step 1: quantitative approach

PRINT-study provided self-report data from the AUDIT-C [13, 43] at baseline, and after 3, 6, 12, 36, and 48 months in a sample of German adults between 18 and 64 years, who were recruited at the municipal registry office in Greifswald, Mecklenburg-Western Pomerania, Germany. Individuals were proactively approached by the study team during their waiting time. The registry office provides access to diverse population segments as German citizens regularly visit for identification documents, vehicle licensing, and residency registration. Of the sample with an average age of 31 years (SD = 11) 56% were women, and 66% were low-risk drinkers. Lifetime abstainers were not included in the sample. More information on the PRINT trial can be found in the protocol [44] and elsewhere [45, 46].

**Fig. 1:**
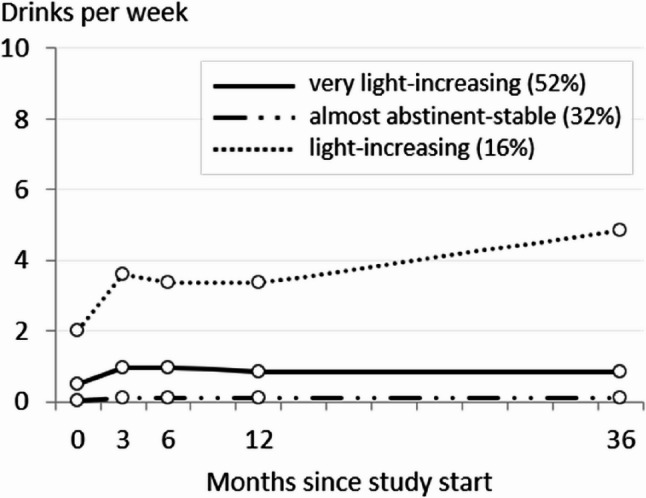
Estimated mean trajectories of drinks per week over 3 years in absence of intervention among low-risk drinkers (*n* = 540), based on preliminary work [42]

Latent class growth modeling was applied to identify distinct classes of individuals with similar trajectories of alcohol use over an observed period of three years (36-month follow-up) [42]. Data from study participants in the control group who were identified as low-risk drinkers by the AUDIT-C [13, 43] at baseline (*n* = 540) were used for the analysis. Low-risk drinking was defined as AUDIT-C scores of 1–3 for women and 1–4 for men [47]. An alcoholic drink was defined as 0.25–0.3 L of beer, 0.1–0.15 L of wine, or 4 centiliters of spirits, each corresponding to approximately 10 to 12 g of pure alcohol. Trajectories were modeled using two growth factors based on indicators representing the number of drinks per week derived from a separate quantity-frequency measure in the last 30 days at baseline, and at months 3, 6, 12, and 36, no AUDIT-C items entered the trajectory models. Time scores were estimated to allow for non-linear trajectories. We followed a systematic approach, testing models with an increasing number of classes (1–5 classes). The optimal number of classes was determined based on multiple criteria: Akaike information criterion (AIC), Bayesian information criterion (BIC), sample-sized adjusted BIC (aBIC), the Lo-Mendel-Rubin adjusted likelihood ratio test (LMR-LRT) as well as theoretical interpretability and class size. As described in more detail elsewhere [42], although AIC and aBIC decreased with increasing number of classes, BIC and LMR-LRT indicated that the three-class model was optimal. Moreover, models with more than three classes produced very small class sizes. Therefore, three latent trajectory classes were identified for the second step of the mixed-method approach: class 1 “almost-abstinent-stable” (*n* = 173, 32%), class 2 “very-light-increasing” (*n* = 281, 52%), and class 3 “light-increasing” (*n* = 86, 16%) (see Fig. 1).

### Step 2: qualitative approach

The qualitative sampling strategy was designed to capture the widest possible diversity of perspectives within these classes. At the 48-month follow-up of the PRINT-study, all participants used for the mixture modeling analysis in step 1 were contacted by telephone and those from the trajectory classes were asked about their willingness to participate in an in-depth interview. From those who expressed interest (class 1: *n* = 51; class 2: *n* = 59; class 3: *n* = 24), we initially drew a random sample of ten individuals from each class, stratified by gender and by three predefined age groups (years of age: 21 to 30, 31 to 45, and 46 to 68). To preserve heterogeneity in age, gender, and class membership, we applied iterative “replacement sampling” from the original pools within each class.

In total, 55 individuals received an invitation including study information by post or email, with up to two reminders in case of non-response. After written informed consent was given, an interview appointment was arranged. All study participants received a voucher worth 50 euros as compensation for their participation in the interview. Ultimately, 22 interviews were conducted (40% of *n* = 55) with ten individuals from class 1, seven from class 2, and five from class 3. The final sample sizes reflect both differential response patterns and thematic saturation. Replacement sampling enabled us to recruit additional individuals from each group, if necessary. However, with regard to class 3, the limits of replacement sampling were reached, once the pool of potential participants who had consented to be contacted for an interview had been exhausted. Thus, the final subsample sizes were ultimately determined by actual participation patterns and the saturation-based stopping criterion.

Data saturation was systematically assessed through parallel analysis. After every three to four interviews, the coding team (M.Z., M.B., S.V.B.) reviewed the emerging themes and determined if new insights were still being generated. Saturation was considered to have been reached when the final interviews yielded no new codes and confirmed the existing coding scheme. The research team (M.Z., A.S., S.B.) regularly discussed the findings from the interviews and data saturation in weekly meetings. As is standard in qualitative research, the goal was depth and breadth of perspectives, not statistical representativeness.

Participants in the almost-abstinent-stable class were not lifelong abstainers; they had consumed alcohol at baseline and maintained minimal use over the 4-year follow-up. At the time of interview, some were current abstinent (e.g., due to pregnancy) or consuming very infrequently, but all had drinking experience and could reflect on drinking motives. As these interviews also provided valuable insights from current non-drinkers, they were retained in the sample, and additional participants from class 1 were recruited for an interview to also capture the perspectives of those still meeting the “almost abstinent” profile. Despite variations in current abstinence status, participants in this class shared similar low-consumption trajectories over the 4-year period, justifying their treatment as one coherent group.

Data were collected from April to June 2022 by two researchers (M.Z., A.S.) and two female research assistants (M.B. and S.V.B.) trained or experienced in qualitative research methods. A semi-structured interview guide (see Supplement) with 15 open-ended questions was developed by three researches (M.Z., S.B., A.S.). The interview questions covered experiences, opinions, and values in relation to the alcohol drinking patterns of the study participants. Four pilot interviews were conducted to test the interview guide and practice the interview situation. After that, the content of the interview guide was adapted. The pilot interviews were not included in the final data analysis.

All interviews were conducted as one-to-one conversation via company mobile phone, on the premises of the Institute and Policlinic of Occupational and Social Medicine in Dresden, Germany. As part of the interview process, the interviewers periodically restated the participants’ responses in their own words to verify whether their understanding was accurate and to avoid potential misunderstandings (participant validation). After each interview, all irregularities were recorded in a brief protocol. The interviewees were not contacted for follow-up questions after the interview, and did not provide any feedback on the results.

Interviews were audio recorded and transcribed verbatim by two research assistants (M.B., S.V.B.). The transcripts were not returned to the interviewees for comments or corrections. Transcripts were anonymized and controlled for accuracy by reading and checking the text by a co-worker who had not transcribed the interview (dual control principle).

We used MAXQDA software (Version 2020) [48] for content-structuring qualitative content analysis according to Kuckartz & Rädiker [49], focusing on descriptive categorization and systematic coverage of the data. This method is designed to produce transparent, replicable categories grounded in participant responses. The initial categories were formed deductively based on the themes of the interview guide. These categories were then expanded inductively by analyzing the transcripts.

The data were analyzed by one researcher (M.Z.) and two trained research assistants (M.B., S.V.B.). Each interview was processed by two coders. Unclear interview quotations and divergent viewpoints of the coders were discussed to create a consistent coding scheme and minimize the impact of researcher’s subjectivity. The multidisciplinary team of authors included researchers from the fields of psychology and health sciences. The authors had varying levels of prior experience in alcohol research and qualitative analysis. This diversity of backgrounds provided different perspectives on interpreting the data. During team meetings, the authors discussed their interpretations iteratively to identify and challenge assumptions, thereby reducing potential bias. We have followed the “Consolidated criteria for REporting Qualitative research” (COREQ) guidance for reporting qualitative approach [50], the completed checklist is provided as supplementary material.

The interview quotes included in the publication were translated from German into English. Insertions that did not contribute to the content, and features of spontaneous speech, such as stuttering, repetitions, self-corrections, filler words, and punctuation marks, were removed to improve the reading flow. Otherwise, we tried to stick as closely as possible to the original statements. The names of the interviewees have been removed for data protection reasons.

### Step 3: integration of results

MAXQDA was used to link the qualitative and quantitative data. This allowed us to analyze the similarities and differences between the identified interview themes in the trajectory classes by noting the frequency with which the themes were mentioned in the transcripts. The latent trajectory classes served as a basis for sampling, ensuring the inclusion of individuals with various long-term drinking patterns in the qualitative phase. This approach increased the likelihood of capturing diverse motivational structures that might not have emerged from a convenience or homogeneous sample.

## Results

### Participant characteristics

Twelve women and ten men participated in the qualitative study (see Table 2). The mean duration of an interview was 40 min (SD = 14).

**Table 2:** Socio-demographic information of the participants (*n* = 22)

Characteristic	*n* (%)
Sex	
Female	12 (55%)
Male	10 (45%)
Age	
21–30 years	11 (50%)
31–45 years	5 (23%)
46–68 years	6 (27%)
Alcohol use latent trajectory class	
Almost-abstinent-stable	10 (45%)
Very-light-increasing	7 (32%)
Light-increasing	5 (23%)
Audit-C-Score	
0	2 (9%)
1	6 (27%)
2	7 (31%)
4	3 (14%)
5	3 (14%)
6	1 (5%)
Smoking status	
Never smoker	19 (86%)
Former smoker	1 (5%)
Current daily smoker	2 (9%)
Highest qualification	
9 or less years of school	0
10 to 11 years of school	5 (23%)
12 or more years of school	17 (77%)
Current employment or education status	
Full time job	8 (36%)
Part time job	3 (14%)
In university	5 (23%)
In vocational training/apprenticeship	2 (9%)
Other, e.g., in retirement or parental leave	4 (18%)
Partnership	
Yes	19 (86%)
No	3 (14%)
Total	22 (100%)

Variables were assessed at the time of the interview, except for school education (baseline assessment of the PRINT study) and alcohol use latent trajectory class (repeatedly measured over three years)

### Coding scheme

The final coding scheme encompassed following main themes: alcohol use patterns throughout life; drinking motives; reasons, barriers, resources, and strategies for situational abstinence; possible influences and effects on consumption; thoughts on risky drinking and prevention. The coding process resulted in 147 codes within the scheme. This paper presents a selection of the most important and relevant ones: drinking motives, e.g., health-related beliefs, as well as reasons, barriers, resources and strategies for situational abstinence (see Table 3).

**Table 3:** Excerpt of the final coding scheme

Categories	Coding definition	Anchor example including information on study participants (sex, latent trajectory class)
Drinking motives		
Enhancement	Alcohol was consumed for pleasure, excitement, to enhance mood or enjoy the taste.	I would miss being on holiday in a beautiful wine region, swirling a glass of wine, enjoying the view, tasting the local wine. (female, light-increasing class)
Social	Alcohol was consumed as a part of social activities, such as social gatherings, or to ease socialization.	Sometimes I wish I could give up alcohol completely, […] But when we celebrate a birthday with everyone sitting together in a cozy atmosphere having a drink, I treat myself to a shandy. […]. I think it is the social aspect that makes me not want to drink mineral water. (female, very-light-increasing class)
Conformity	Alcohol was consumed because of perceived social pressure or a fear of being left out, as well as because of cultural norms or traditions.	I have learned that toasting is done with alcoholic drinks and not with coffee or water, as it is an unwritten rule of good manners. (female, almost-abstinent-stable class)
Coping	Alcohol was consumed to avoid disturbing thoughts, troublesome feelings, or personal problems.	I can imagine that alcohol might sometimes serve as a reward. For example, when I had a glass of sparkling wine last Friday, I had just finished a stressful week at work, and I enjoyed the relaxation it provided, perhaps even more than I would have experienced if I had stayed sober. (female, very-light-increasing class)
Health-related beliefs	Alcohol was consumed because health benefits were anticipated.	I read a bit about healthy eating and [red wine]^a^ was recommended because of the ingredients. So, I thought, if there is ever an occasion, I can have a glass of red wine. […] It said that it is really not harmful [to drink] from time to time. (female, almost-abstinent-stable class)
Situational abstinence		
Reasons	Underlying considerations that cause individuals to abstain from drinking alcohol in certain contexts or situations, but not to the point of striving for general abstinence.	If you do not enjoy riding a motorcycle, you will never get a license and buy a motorcycle. It is the same for me with alcohol. I get no benefit from drinking alcohol. It is just irrelevant. (male, almost-abstinent-stable class)
Barriers	Psychological, social, and cultural factors that prevent or discourage individuals from abstaining from alcohol in certain situations, even when they have reasons or intentions to do so.	In some social situations, it can be difficult to admit that you do not drink alcohol at all because it may not be well accepted. This is similar to the problem vegans face. In order to not stand out and fit in with the group, I would drink a small amount of alcohol. (female, very-light-increasing class)
Resources	Personal or social assets that enable individuals to successfully abstain from alcohol.	I simply stated that I did not want to drink alcohol and that I could enjoy myself without it. This was accepted by everyone. (female, almost-abstinent-stable class)
Strategies	Concrete behavioral approaches and techniques that individuals employ to avoid or refuse alcohol.	I was often put in situations where I felt the need to justify myself. Then I said things like: I am on a medication, I do not feel well today […] I often lied because I simply had no appetite for alcohol. (female, almost-abstinent-stableclass)

^a^Additions for a better understanding of the interview statement are indicated by words in square brackets

#### Drinking motives

The interviewees’ narrative accounts of their motives were often fragmented, hesitant, or contradictory. Several interviewees seemed to reflect on their reasons for drinking for the first time and struggled to articulate them. The following excerpts illustrate habitual, automated drinking patterns or unreflective drinking routines:

You always have beer on hand for visitors, so you can offer them something. During the Corona period, there were not many visitors. The problem was, the beer would go bad. So, you drink a beer to empty it. [...] It does not have to be pure beer, perhaps it could be a mixed beer. The taste of the beer is mostly masked by the lemonade or other additives, such as fruit. The beer is only subliminally present, it is no longer pure. But why I choose a mixed beer instead of soda, I cannot answer right now. I do not know. (male, light-increasing class)

I have ten or fifteen bottles of wine here. [...] It is just sitting around because it was on sale. [...] You have drinks, but you do not drink them. Why? Because you have the space. [...] There is not a special reason or a mood. I can drink a glass of water too. I do that sometimes, too. But yes, togetherness for two is also something! […] We will finish the bottle of wine over the course of the week. [...] No, no, no. Let me put it this way: I do not crave wine or beer. (male, very-light-increasing class)

Additionally, the interviews revealed that motives were rarely fixed. They could shift between motives during a single occasion. For example, one man described attending a party:

I think party friends, they keep you young. It is nice to have this balance, and I think alcohol helps everyone get in the mood, make a good party even better, and make a bad party more bearable. [...] I mean, because sometimes you are just grateful to be invited. [...] Sometimes I leave early, but when I am at a bad party and there is free beer, it is hard not to have one or two. (male, light-increasing class)

Here, reference to enhancement (“making a good party even better”) blends into conformity (“drinking because others did and feeling grateful to be invited”) and shifts toward coping (“making a bad party more bearable”) depending on situational context. This interplay illustrates the dynamic and co-occurring nature of drinking motives. To structure these heterogeneous comments, we draw on the established four-factor model of drinking motives.

##### Enhancement

Enhancement, characterized by drinking to amplify positive emotions and experiences, focused on appreciating taste and enhancing specific pleasurable moments. Most frequently, interviewees reported drinking low-percentage alcoholic beverages such as beer or wine during weekend evenings or other leisure moments, explicitly linking their alcohol use to states of well-being and positive mood. The importance of taste and quality was evident in their willingness to invest in premium products for unique experiences. As one interviewee explained:

I am not an expert, but a good Rothschild is worth enjoying. I prefer not to buy or to drink cheap booze. (male, very-light-increasing class)

Interviewees described high-quality alcohol as something to be appreciated, requiring careful selection and mindful consumption. The aversion to "cheap booze" showed a preference for quality over quantity. These preferences were particularly evident in dining contexts. Interviewees often described wine as an essential part of a great meal, where alcohol enhances the overall experience of taste and smell:

I feel very exquisite when I choose a wine at a restaurant. It shows that I am familiar with the subject and can contribute to the conversation about popular trends in the fine dining. [...] To me, going out for a fine meal and not ordering wine would be an insult to the food. It would show that I do not know anything about gourmet delights. (female, almost-abstinent-stable class)

This comment illustrates how enhancement motives can be anchored in specific contexts and sensory experiences. In this case, the restaurant emerges as a ritualized setting where alcohol use acquires symbolic meaning and follows a social code, as wine is expected to accompany food. Ordering wine with food was not only framed as enhancing the dining experience, but also as demonstrating cultural knowledge and sophistication, signaling status and group belonging. At the same time, the interviewee emphasized that abstaining would be perceived as a faux pas (“insult to the food”), risking social disapproval. Thus, this behavior extends beyond personal pleasure to include impression management and the avoidance of social loss.

##### Social

Beyond individual enjoyment, interviewees consistently emphasized the social dimension of drinking. This was the only theme that emerged in every single interview, highlighting its central role. Alcohol often gained meaning through its ability to foster connection and conviviality, as one interviewee described:

Yes, it relaxes you. It makes conversations possible. It allows you to unwind, even with new people, and get to know them at a party or in a bar. When you meet new people, it is easier to start a conversation. (male, light-increasing class)

Most interviewees emphasized the importance of socializing, often noting that they would not drink alone. Alcohol was associated with occasions such as birthdays, family gatherings, and evenings with friends. Joint consumption contributed to creating a “cozy atmosphere” and signaling togetherness. This implied that everyone was expected to align their drinking habits, if not literally match the same amount.

Some interviewees stated that they drank more than usual on special occasions when alcohol was abundant or free. Rather than representing a distinct motive, this indicates that availability is a situational facilitator. The interviewees did not actively seek out free alcohol; rather, they tended to drink more when drinks were readily available or did not incur an additional cost. Thus, availability appears less as an independent driver of consumption and more as a catalyst that amplifies existing social motives.

##### Conformity

In our interviews, conformity motives clustered around two distinct forms: perceived situational peer pressure and the influence of cultural traditions. Both reflect a wish to avoid standing out or remaining a part of a culture, family or community, rather than to seek enjoyment or enhancement. On the one hand, several interviewees stated feeling obliged to drink alcohol in certain social situations, particularly at celebrations. Some of them believed that declining an alcoholic drink could be considered rude or make them appear out of place. Sometimes, a negative reaction was anticipated even when no actual pressure was exerted. As one interviewee explained:

Sure, there is always someone who says, "No, I do not drink," […]. I have noticed this more frequently among my friends and relatives lately. But at the celebrations so far, though, it was like: The others drink, so you just drink too. I cannot attribute this to taste, because alcohol does not necessarily taste good to me. It is not because I become more relaxed through it either [...] You do not want to stand on the sidelines because you are not drinking. You still get looked at strangely. (male, light-increasing class)

The comment illustrates how the fear of social marginalization can prompt people to drink alcohol. The statement “You still get looked at strangely” provides insight into “drinking to fit in,” which may reflect a level of social anxiety that drives engagement with alcohol.

On the other hand, some comments pointed to long-standing cultural traditions. These traditions were often introduced in childhood and became strongly associated with family rituals and regional identity, such as toasting with sparkling wine on New Year’s Eve, toasting at a youth ceremony or drinking young wine at harvest time. One interviewee described:

That is more like a traditional story, the season of Federweißer [young wine]. My parents taught me about this tradition. I get a bottle of young wine, make an onion cake, and keep the tradition alive. [...] I would say the onion cake must be eaten with young wine because the two go perfectly together. [...] It is deeply rooted in my Palatinate culture. [...] Today, I was still eating the onion cake, but the wine was already gone, and the cake still tasted good! It is actually nonsense to say the food only tastes good with wine. But I was taught differently in my family. […] We had a winery, and my father made a living selling wine. Of course, he always emphasized the tradition of sociable wine drinking. (female, almost-abstinent-stable class)

Here, conformity operates through internalized cultural norms. Although the interviewee recognized the randomness of pairing food and wine (“actually nonsense”), the practice remains meaningful and continues to influence the behavior. At the same time, the statement reveals a certain ambivalence, marked by the tension between critical reflection and continued adherence. This highlights the stable and enduring nature of cultural conformity, as opposed to the more situational dynamics of peer pressure.

##### Coping

Most interviewees explicitly distanced themselves from using alcohol to manage negative emotions or stress. As one of them explained:

I make sure that alcohol is a social and positive experience. I do not use it to feel better or to forget, because... Well, my mother was an alcoholic, and I saw where that led when alcohol is used to forget or to drink away bad feelings. I set a rule for myself that I will not use alcohol for that purpose. (female, light-increasing class)

This strong rejection of coping motives suggests awareness of alcohol risks and a deliberate choice to confine drinking to positive and social situations. In some cases, this distancing was rooted in personal or family experiences with problematic drinking, which reinforced protective boundaries against emotion-oriented alcohol use.

We identified two quotes that could be tentatively interpreted as touching on coping motives. One of them was introduced earlier in the context of co-occurred motives (see above). The interviewee described how alcohol could make a “bad party more bearable”, which can be seen as a situational response to social discomfort rather than a consistent coping pattern. The second quote involved an interviewee who described using alcohol as a reward after a demanding week at work (see Table 3), explicitly noting finding it more relaxing than staying sober. Thus, coping did not emerge as a recurring theme but rather as an occasional nuance within a broader pattern of positive, social, or habitual drinking.

##### Beyond the four-factor model: health-related beliefs

In addition to the four established motives, a smaller number of interviewees referred to health-related reasons for drinking alcohol. One reported drinking a mixed beer after exercise, believing it replenished electrolytes and aided rehydration. Another one explained that red wine use can help maintain blood vessels healthy. One interviewee mentioned that is well known that moderate alcohol use can be beneficial and well tolerated by the body. Finally, one interviewee preferred beer over soft drinks because the latter were perceived as less healthy due to their high sugar content. He particularly clearly articulated the comparative health logic:

Soda often has a negative connotation because of its high sugar content. It is unclear what other ingredients it contains. According to the German Purity Law, beer can only contain hops, malt, and water. Although it contains alcohol, beer might actually be healthier than soda. (male, light-increasing class)

This comment reveals how the interviewee thought about subjective risk calculations. He positioned beer as potentially healthier than alternatives by weighing different risk factors, such as sugar content and unknown additives. Notably, he used the “German Purity Law” as a framework for assessing product safety, suggesting that health-related drinking decisions are not based solely on medical evidence, but also on subjective, experiential, and deliberative judgments. Furthermore, describing beer as “healthier” than soda not only neutralizes one’s alcohol use, but also gives it a positive charge.

#### Situational abstinence

In this study, we understand situational abstinence as the practice of avoiding alcohol in certain situations or contexts rather than abstaining altogether. In order to gain a better understanding of this practice, we examined the reasons interviewees gave for abstaining, the barriers they encountered, the personal and social resources they could draw upon, and the strategies they employed in such situations.

##### Reasons

When asked about situations in which they abstain, interviewees described various contexts that fell under a few main motives. A prominent theme was avoiding health-related consequences. The interviewees reported abstaining to prevent immediate impairments, such as hangover, nausea, and feeling unwell. A smaller number of interviewees also mentioned concerns about long-term health risks to the body and mind associated with alcohol. Other frequently cited reasons were safety concerns and taking responsibility, e.g., never drink and drive, responsibilities toward others (e.g., children or friends), duties the next day (e.g., appointments or work). Finally, a desire for control was reported when those affected feared saying or doing things under the influence of alcohol that they would regret. These reasons were often shaped by direct and vicarious experiences. Observing problematic drinking in others or reflecting on their own past overuse shaped their awareness of vulnerabilities and informed abstinence in specific contexts. For instance, family models mattered both as deterrents (parental overuse) and as positive influences (non-drinking at home).

Beyond these mentioned reasons to abstain, two additional patterns emerged. The most frequently mentioned point was that the interviewees simply did not consider alcohol to be necessary. They emphasized not missing it and being able to enjoy themselves without it. One woman reported a simple absence of drinking motivation: “I can have fun without alcohol” (female, light-increasing class). Others rejected alcohol as a matter of social autonomy or independence from group norm, resisting expectations to drink merely because others expected it. As one woman mentioned:

When people say, "Now you really have to drink something," I say, "No, why should I?" It is just a birthday. That does not mean I have to drink alcohol. I will toast with orange juice, for example, while others might say, "Well, okay, then I will have a drink." (female, almost-abstinent-stable class)

##### Barriers

Despite various reasons for situational abstinence mentioned in the interviews, we identified barriers that make sustained abstinence challenging. The most prominent barrier was interviewees’ perception of the long-term health risks associated with their current alcohol use. Most of them emphasized that they saw no reason to reduce their alcohol use, because they did not expect any negative consequences.

At the moment, I see no need to give up alcohol because I am doing well. If I compare myself with other people around me, I am the one who drinks the least. (female, light-increasing class)

For some, abstinence even seemed counterproductive. Two interviewees perceived abstinence as a self-imposed restriction that might trigger the opposite effect:

I do not like that kind of absolutist thinking. If I told myself not to drink alcohol under any circumstances, I would be more inclined to drink. (female, almost-abstinent-stable class)

Beyond individual reasoning, cultural and social factors emerged as powerful barriers. Most interviewees reported the strong normalization of alcohol in Germany. Drinking was described as an integral part of celebrations, festivals, and social gatherings, while abstaining requires an explanation. This reflects cultural norms that embed alcohol into traditions, as well as descriptive norms that indicate widespread drinking behavior. Some interviewees reported experiencing social pressure related to non-drinking. They mentioned they were questioned about their decision to abstain and sometimes pressured to change their minds.

Something like, “Come on, have a drink! You are the only one not drinking. One drink is okay, even if you are driving.” (female, very-light-increasing class)

This pressure illustrates injunctive norms questioning their deviation from the expected drinking behavior. While some gave in to avoid standing out, others expressed a deeper concern that sustained abstinence might result in social ostracism.

I am really afraid to stop drinking altogether. I am afraid that others will resent me, misunderstand me, think I am a spoilsport, lose sympathy for me or get angry with me. (female, almost-abstinent-stable class)

Given my medical background, I am familiar with the research on the so-called J-curve and I understand that complete abstinence from alcohol is not necessarily the healthiest option. Nevertheless, this is not a reason for me to drink. I just think that, in some situations, at social events, it can be very, very difficult to be upfront about non-drinking at all, because it is not widely accepted - much like declaring oneself vegan. To avoid standing out or drawing attention, I would continue to drink a little. (female, very-light-increasing class)

Such statements suggest that barriers are not limited to situational peer pressure, but include anticipatory fears of exclusion, that inhibits the transition from situational abstinence to sustained abstinence. Comparing abstinence to veganism illustrates how non-drinking is perceived as socially deviant, requiring explanation and justification, while drinking is the default, socially acceptable behavior.

##### Resources

Interviewees described their refusal confidence as rooted in self-determination, willpower, and assertiveness, which they saw as personal resources that had often developed gradually through personal growth. Several interviewees emphasized that once they had firmly decided not to drink, they would remain true to this decision despite external expectations.

Over the course of my life, I have become more self-confident. It is now easier for me to voice my opinion without feeling insecure when others around me have different views. (female, almost-abstinent-stable class)

Beyond personal agency, social resources also played a role. Several interviewees emphasized that their drinking habits were recognized and accepted by their social environment, reducing the need for repeated justifications. Some even described receiving active support from partners who also abstained, which reinforced their choices. These findings reflect helping relationships, in which social support and acceptance from peers and partners reinforce individual choices and facilitate the maintenance of abstinence.

##### Strategies

Though not always framed as deliberate plans, interviewees reported a range of strategies to manage drinking occasions. At the individual level, approximately half of the interviewees described directly declining offered drinks. They emphasized their ability to assertively say “no” when they had firmly decided to abstain. Another common approach was to use non-alcoholic beverages to participate in social rituals without consuming alcohol. As one interviewee described:

I also think that there are more and more good alternatives, such as non-alcoholic beer. I try to bring this up more often because I have noticed that the sense of belonging, I get from holding a beer gives me a better feeling than the alcohol itself. (female, light-increasing class)

At the contextual level, some interviewees actively avoided situations or individuals associated with heavy drinking, such as skipping certain gatherings or keeping distance from peers who drank excessively. Additionally, a few interviewees declined alcohol using socially acceptable excuses, such as having to drive or taking medication.

### Integration of results

Table 4 provides an overview of the most salient themes and their respective frequencies of mention across the identified latent trajectory classes:

**Table 4:** Distribution of the main themes according to the three latent trajectory classes

Main themes	Latent Trajectory Classes and number of interviews in which the statement was mentioned			Analytical Integration
	Almost-abstinent-stable (10 interviews)	Very-light-increasing (7 interviews)	Light-increasing (5 interviews)	
Drinking Motives				
Enhancement	6^b^	5	5	Enjoyment motives were mentioned frequently in each class.
Social	10	7	5	Social motives were mentioned frequently in each class.
Conformity	5	5	5	Conformity motives were mentioned frequently in each class.
Coping	0	1	1	One interview in the light-increasing and one in the very-light-increasing class contained a reference to coping.
Health-related beliefs	1	2	1	Drinking for health benefits was mentioned rarely in each class.
Situational abstinence				
Reasons:				Health-related consequences, safety, and responsibility as reasons for situational abstinence were mentioned frequently in each class. The desire for control and self-determination, as well as no need in alcohol were more often mentioned in the almost-abstinent-stable and very-light-increasing class. The desire for social autonomy was frequently mentioned in the almost-abstinent-stable and very-light-increasing classes but not in the light-increasing class.
Health-related consequences	6	6	5	
Safety	6	4	2	
Responsibility	3	6	3	
Desire for control/Self-determination	5	2	1	
Alcohol not necessary	9	5	1	
Social Autonomy	4	4	0	
Barriers				Individual from all classes frequently reported no need to reduce their alcohol use. Social norms as a barrier for abstinence was perceived in all three classes, while cultural norms were most frequently mentioned in the almost-abstinent-stable class.
No need to reduce	7	6	4	
Cultural norms	9	6	2	
Social pressure	4	6	3	
Resources				Personal resources, such as self-determination and willpower were more often mentioned in the almost-abstinent-stable and very-light-increasing classes than in the light-increasing class. A supportive social environment was frequently mentioned resource in all classes.
Personal	7	5	1	
Social	8	4	4	
Strategies				Declining offered drinks was the most consistently mentioned strategy across classes. The almost-abstinent-stable class reported using non-alcoholic alternatives and avoiding gatherings more frequently. Using excuses was the least common strategy overall.
Declining offered drinks	3	6	3	
Non-alcoholic drinks	5	2	2	
Avoiding gatherings	6	2	1	
Using excuses	3	0	1	

^b^Parentheses contain the number of interviews, in which the respective category was mentioned

#### Drinking motives

In our sample, social motives dominated across all classes, followed by enhancement and conformity motives, while coping motives appeared in only two interviews. This indicates that alcohol was largely associated with social integration and mostly positive experiences rather than managing negative emotions. Every interview in the light-increasing class contained references to drinking for conformity reasons, suggesting that conformity may be a most relevant driver distinguishing the light-increasing trajectory from more stable low-consumption patterns such as almost-abstinent-stable. However, rarely mentioned, health-related beliefs were still cited as a reason to drink, indicating that some interviewees perceived alcohol as potentially beneficial to their health.

#### Situational abstinence

Health-related consequences, safety, and responsibility as reasons to abstain were mentioned frequently across all three classes, indicating a shared awareness of potential risks of alcohol use. The almost-abstinent-stable class, however, stood out for more frequent references to control, self-determination, and the perception that alcohol was simply unnecessary. Social autonomy, defined as independence from drinking expectations, was also raised in this group and in the very-light-increasing class. This suggests that the light-increasing class may be more susceptible to social drinking pressures and less driven by internal autonomy motivations compared to the other classes.

#### Barriers

Interviewees from all classes frequently stated that they saw no need to reduce their alcohol use further, reflecting both their low levels of consumption and the perception that the potential risks associated with their drinking might be negligible. Additionally, interviewees across all groups described experiencing social expectations in drinking situations, such as being asked why they were not drinking or being repeatedly encouraged to join in. The almost-abstinent-stable class most frequently pointed to the omnipresence of alcohol in cultural practices, the general ease of access, and widespread acceptance of drinking. This suggests that those maintaining the most consistent low consumption may be most aware of cultural expectations that make abstinence socially challenging.

#### Resources

With regard to resources, personal characteristics such as self-determination and willpower were reported more often in the almost-abstinent-stable and very-light-increasing classes. This suggests that individuals who maintained the lowest levels of alcohol use tended to rely more strongly on personal resources. Social support, in contrast, was reported in all three groups.

#### Strategies

Declining offered drinks was mentioned across all three classes. Beyond that, the almost-abstinent-stable class reported using non-alcoholic beverages as substitutes and avoiding gatherings where alcohol is consumed more frequently. Excuses were the least common overall, but were somewhat more prevalent in this group. However, these “strategies” were not always described as consciously planned. Rather, they often appeared as habitual or automated behaviors that developed naturally within the social environments of the interviewees.

## Discussion

### Summary of main findings

This longitudinal mixed-methods study explored drinking motives and attitudes toward abstinence among German adults in general population with low-to-moderate alcohol use across different drinking patterns: almost-abstinent-stable, very-light-increasing und light-increasing. While qualitative interviews revealed that social motives dominated across all trajectory classes, coping motives played a negligible role. Interviewees frequently practiced situational abstinence, however, sustained abstinence faced significant barriers, including low risk perception of the own drinking practice, strong cultural normalization of alcohol in Germany, as well as social costs of non-drinking.

Additionally, while low-to-moderate drinkers share many motives and barriers for situational abstinence, subtle but consistent differences distinguish the almost-abstinent-stable and very-light-increasing classes from the light-increasing class. Stronger personal resources, such as self-determination and willpower, and more frequently cited control- and autonomy-related reasons for abstaining appear to mark trajectories of more stable low use, whereas reliance on social norms characterize those with gradual increases. Overall, lower-consumption groups appeared to interact with alcohol in a more deliberate and self-directed manner. In contrast, the light-increasing group’s abstinence appeared to be more passive, emerging through social alignment and situational factors.

### Drinking motives among low-to-moderate drinkers

Even after taking time to reflect, some of the low-to-moderate drinkers in our study had difficulty identifying reasons for their alcohol use. Some accounts of decision-making were marked by ambivalence, suggesting unreflective, habitual responses to social cues and environmental availability. According to motivational theory, decisions to drink often occur automatically without people being fully aware that they have decided to drink or of the influences shaping their decision [14, 22]. In such cases, motives may not be explicitly stated, yet they still influence behavior and become apparent through how individuals describe their drinking situations and practices. These patterns can be meaningfully interpreted within established motivational frameworks, such as the four classic motive dimensions.

In our study, individuals most frequently reported drinking for *social reasons* and avoiding drinking alone. This is in line with previous findings showing that social motives are common in different samples and associated with moderate and infrequent drinking particularly in social contexts [15, 16, 22–24],, whereas they are negatively associated with drinking at home or drinking alone [16]. In this sense, social motives frame drinking less as an individual choice and more as part of shared routines and occasions.

Although *enhancement motives* are generally more strongly linked to heavier drinking [16, 20, 23, 26, 27, 51], they also appeared in our sample. However, among low-to-moderate drinkers these motives took a different form that in populations such as young adults who drink on weekends to have fun and get drunk [22, 27]. In our data, enhancement was linked to celebrating special occasions or appreciating taste, rather than seeking intoxication or thrills.

Whereas enhancement reflected the pursuit of pleasure, *conformity motives* reflected the adjustment of drinking behavior to external expectations. Conformity is often linked to avoiding social rejection or yielding to peer pressure [15, 16, 22, 24, 28] and typically associated with moderate rather than risky drinking in specific contexts [15, 16, 24]. In our interviews, conformity also encompassed cultural expectations. Cultural norms and traditions represent a internalized form of conformity, as drinking behaviors being culturally regulated, justified and prescribed [52]. Unlike direct peer pressure, these influences operate more subtly, positioning drinking as a way to affirm social identity and belonging through shared norms and values. Cultural and social contexts shape drinking behaviors and influence perceptions and judgments about what constitutes appropriate consumption [52–55]. This pattern is reflected in our sample, where interviewees sometimes maintained traditions, they could not even explain or openly questioned.

In contrast to these social and cultural influences, *coping* played a negligible role in our sample of individuals with low-to-moderate alcohol use. Several interviewees explicitly rejected such motives as incompatible with their understanding of “responsible drinking.” This suggests that alcohol use is rarely employed as a strategy to manage negative emotions among low-to-moderate drinkers. This is consistent with prior research showing that coping motives are rarely associated with low alcohol use [16, 20, 21, 23, 26, 51, 56].

Our findings therefore suggest that among individuals with low-to-moderate alcohol use the established motivational structure is reflected. However, in this group it is characterized by a strong emphasis on social motives and occasional enhancement. This aligns with earlier research showing that moderate drinkers report fewer motives overall than heavier drinkers, while still endorsing social and enhancement motives [56].

Beyond the traditional motivational framework, we found that health-related beliefs shaped how interviewees reasoned about alcohol use. Avoiding health consequences was a frequently mentioned reasons for situational abstinence, but this did not translate into permanent abstinence. At the same time, some interviewees supposed positive effects of alcohol and downplay its potential harm, for example by contrasting it with sugary drinks. Such reasoning suggests that interviewees may not fully recognize that even small amounts of alcohol increase disease risk compared to complete abstinence.

Furthermore, we identified impression management as an additional motivational dimension. Impression management refers to the process used to monitor and control how one is perceived and evaluated by others [57, 58]. In our sample, alcohol use was sometimes employed to signal cultural knowledge, sophistication, or social competence. This aligns with broader evidence that impression management shapes social behavior by influencing treatment by others, strengthening self-concept, fulfilling belonging needs, and optimizing social exchange [59, 60].

Finally, our findings underscore that motives rarely operate in isolation. Instead, they can co-occur or alternate, particularly in social contexts. Coping and enhancement motives, as well as social and conformity motives, have been shown to reinforce each other, while social motives were positively associated with enhancement motives over time [61]. Such complex motivational constellations highlight the potential value of tailored interventions, as proposed by recent protocol studies examining motive-specific alcohol interventions [62].

### Social costs of non-drinking

Low-to-moderate drinkers in our study were reluctant to abstain from alcohol entirely, even though the reduction in absolute quantity of alcohol consumed itself would be small. One reason might be the anticipation or actual experience of negative reactions in case of non-drinking, e.g. challenges to the decision not to drink. Some interviewees made excuses or drank alcohol even though they did not want to, just to avoid the social costs of non-drinking.

Qualitative evidence from other countries support the notion that non-drinking may violate social norms [32, 63–65], probably even more so in a high-consumption country as Germany, in which alcohol is regarded as a cultural asset [66]. Rather than being viewed as positive role models who do something good for their health, non-drinkers may be perceived as a threat to others’ enjoyment and their personal choice to consume alcohol [32, 67]. The anticipation of being judged, confronted, or disapproved when refusing an alcoholic drink in public [33, 63, 64, 68], the experience of peer pressure [69] or the fear of missing out [70] might be barriers to non-drinking and ultimately, a healthy lifestyle. Efforts and strategies to prevent alcohol-related harm should be conscious of strong injunctive drinking norms [71, 72] and the effect of individuals’ pursuit of conformity and social identity [73, 74] against the societal reality in which the act of non-drinking may be viewed as a behavior that is characteristic of an outgroup. The unfavorable social consequences of non-drinking, some of them reported in the interviews presented above, could go so far that they even resemble processes of stigmatization of non-drinking [30, 32, 63, 75, 76]. People who contemplate the idea of non-drinking (either temporarily or permanently) may face stereotypical, derogatory, or even discriminatory reactions from others, potentially undermining their intention not to drink.

### Protective behaviors in low-to-moderate drinkers

In our sample, low-to-moderate drinkers reported that they could easily control their alcohol use. Some interviewees mentioned various protective thoughts and behaviors that align with both Protective Behavioral Strategies frameworks [77–79] and change processes from the Transtheoretical Model [36–38]. Self-liberation, or the commitment to act in accordance with one’s own decision despite social pressure, was evident when interviewees declined offered drinks. Counterconditioning, or replacing problematic behaviors with healthier alternatives, appeared when individuals switched to non-alcoholic beverages to continue socializing without drinking alcohol. Stimulus control, which involves actively modifying one’s environment to reduce triggers for drinking, was demonstrated by avoiding heavy drinkers or events where excessive drinking was expected. Finally, the concept of “helping relationships,” or seeking and using social support, emerged when interviewees described receiving encouragement or understanding from friends and family regarding their drinking choices.

Previous studies have shown that protective strategies can support low-risk drinking [55, 79–81]. Although such patterns were evident in our data, interviewees did not describe them as deliberate strategies. Rather, they emerged as natural, automatic responses in the context of low-to-moderate alcohol use. Among the almost-abstinent-stable, self-liberation stood out as particularly salient in this group, as the individuals remained firm in their choice and resisted external influence, highlighting self-determination and social autonomy as important personal resources. While self-liberation is primarily a cognitive-affective process, our data suggest that it manifests behaviorally through concrete refusal actions and becomes part of one’s drinking identity.

### Psychological reactance

Finally, some low-to-moderate drinkers viewed abstinence as a form of “self-imposed prohibition” that could lead to increased cravings for alcohol. When individuals are advised to employ specific strategies to reduce their alcohol use, they may experience a feeling of being patronized and criticized, potentially resulting in the opposite of the intended effect [80, 82, 83]. This aligns with the concept of psychological reactance, in which individuals resist perceived threats to their decision-making autonomy [82, 83]. Our findings therefore caution that overly prescriptive interventions may be counterproductive and instead underscore the importance of approaches that respect autonomy and foster self-directed change.

## Strengths and limitations

Building on our previous trajectory classes analysis [42], which identified three longitudinal patterns of alcohol use among a sample of the German general population, the qualitative approach purposely sampled individuals across these classes. This sequential mixed-methods design enabled us to capture variation in lived experiences within empirically derived drinking pathways and to move beyond a convenience sample. While the quantitative analysis mapped the structure and stability of the trajectories, the qualitative interviews provided insight into the motives, barriers, and contextual factors that shape them. Our study addresses an important and underexplored topic by investigating motives for (and against) alcohol use as well as barriers and resources to abstain entirely among current abstainers and low-to-moderate drinkers. This focus is highly relevant and timely in the context of growing interest in population-wide alcohol reduction strategies. The focus on adults with low-to-moderate alcohol use from general population, who have rarely been the focus of research, were other strengths of our study.

While data saturation and sampling strategy guided our sampling process, our study also demonstrates high informational power [84] across several key dimensions: First, our narrow, well-defined study aim increased specificity. Second, our sample was highly targeted and relevant, drawn from a longitudinal cohort that we had followed for four years with repeated assessments, ensuring deep familiarity with participants’ drinking patterns. Third, our study was grounded in established theoretical frameworks, including Cooper’s drinking motives model [15] (informing our deductive categories), and concepts from the Transtheoretical Model [36, 37] including self-efficacy and barriers/resources. Fourth, we achieved high-quality interview data through theoretically grounded interview guides, pretesting, and interviewer training. Fifth, we employed systematic content-structuring qualitative content analysis [49], and followed the COREQ guidance [50] for reporting qualitative approach. Although some trajectory classes had small numbers of participants, the theory-informed approach and the richness of the data obtained were sufficient to identify meaningful patterns and achieve our study objectives. Additionally, the qualitative approach prioritizes depth of understanding over statistical generalizability.

Nevertheless, some limitations need to be acknowledged. First, the study was conducted in a specific cultural context (Mecklenburg–Western Pomerania) in a country with high per capita consumption (Germany) [40, 85], so the results may not be fully transferable to other cultural or social contexts. Second, the sample was recruited from a single municipal registry office in Greifswald, a city with a high proportion of university employees and students. Registry office–based recruitment is known to be selective [46], and different socioeconomic groups may visit the registry office with varying frequency, which could lead to the under- or overrepresentation of certain population subgroups. Third, in the qualitative part of the study, potential for interviewer bias, participant self-reporting bias, and effects of social desirability cannot be ruled out. Fourth, while our analysis relied on trajectory classes as the primary analytical framework, (e.g., direct comparison of abstainers vs. light vs. moderate drinkers) could yield different insights in the motivational distinctions between abstainers, low and/or moderate users. Fifth, in latent class growth modeling, individuals are assigned to a specific latent class based on their highest posterior class-membership probability. Because we treated class membership as an observed grouping variable, some individuals may have been misclassified, potentially reducing the precision of comparisons between trajectory classes.

## Conclusion

This longitudinal mixed-method study provided valuable insights into the drinking motives and attitudes towards abstinence in individuals with low-to-moderate alcohol use in Germany. Our findings show that, even among low-to-moderate drinkers, alcohol use is shaped more by social expectations, routines, and cultural normalization than by individual need. Although motives such as enhancement and conformity play a role, coping was virtually absent. Many participants struggled to articulate reasons for drinking, pointing to the habitual and often unreflective nature of alcohol use. At the same time, situational abstinence was common, though sustained abstinence faced structural and social barriers. Notably, comparing trajectory classes reveals that abstinent-stable and very light drinkers often frame their decisions in terms of autonomy and self-control. In contrast, light-increasing drinkers seem more influenced by social alignment. These insights underscore the importance of considering not only consumption levels, but also underlying motivational factors when designing population-level alcohol reduction strategies.

## Supplementary Information

Supplementary Material 1

Supplementary Material 2

## Data Availability

The datasets generated and/or analyzed during the current study are not publicly available due data protection regulations but are available from the corresponding author on reasonable request. Interview transcripts are available as anonymized data in German only.
