# Supplementary material for: Exploring the motives for drinking less alcohol and attitudes towards abstinence in individuals with low-to-moderate alcohol use – a mixed-methods study
Source: BMC Public Health. 2026 Jan 7;26:355. doi: 10.1186/s12889-025-26015-7 (PMC12849377; doi:10.1186/s12889-025-26015-7)
Supplement: Supplementary file 2 — Supplementary Material 2 [file 12889_2025_26015_MOESM2_ESM.docx]

# SUPPLEMENT

## Interview guide

| Icebreaker: Welcome, short introduction |
| --- |
| Formal aspects: Study aims, timeline, procedure, explanation of participant selection, further information, consent to audio-record the interview |
| Introduction to the topic “Trajectory Class”: Discuss the interview participant’s alcohol use over the past three years based on self-reported data. |
| 1. What do you notice when you look at this graph of your alcohol use? 2. Do you recognize your own behavior? (If not, why do you think that is?) 3. To what extent has your perception of alcohol use changed over the course of your life? |
| Resources and barriers for drinking less alcohol: Discuss personal beliefs that are important when drinking alcohol. |
| 1. Can you describe a typical day when you drink alcohol? On what occasions do you usually drink? 2. What mood are you usually in when you drink alcohol? 3. In which situations do you refrain from drinking, even though others around you are drinking? 4. What has particularly influenced your view on alcohol use? 5. What personal advantages and disadvantages do you associate with drinking alcohol? 6. To what extent do you consider the consequences your alcohol use might have? 7. What role does alcohol play in your social environment? Who supports or opposes drinking? 8. How do you respond when someone asks why you don’t drink? 9. How confident are you that you could decline a drink if someone offered you one? Please rate your confidence on a scale from 1 (“not confident at all”) to 10 (“very confident”). Why did you choose that number instead of a higher or lower one? |
| Prevention: Discuss what could or should be done to motivate people to drink less alcohol. |
| Conclusion – summary and reflection:   1. Is there anything else you’d like to add about alcohol use that we haven’t discussed yet? 2. Do you have any questions?   Thank the participant and say goodbye. |
